# Acquisition of Chemoresistance in Gliomas Is Associated with Increased Mitochondrial Coupling and Decreased ROS Production

**DOI:** 10.1371/journal.pone.0024665

**Published:** 2011-09-09

**Authors:** Claudia R. Oliva, Douglas R. Moellering, G. Yancey Gillespie, Corinne E. Griguer

**Affiliations:** 1 Division of Neurosurgery, Department of Surgery, University of Alabama at Birmingham, Birmingham, Alabama, United States of America; 2 Department of Nutrition Sciences, University of Alabama at Birmingham, Birmingham, Alabama, United States of America; 3 Center for Free Radical Biology, University of Alabama at Birmingham, Birmingham, Alabama, United States of America; Florida International University, United States of America

## Abstract

Temozolomide (TMZ) is an alkylating agent used for treating gliomas. Chemoresistance is a severe limitation to TMZ therapy; there is a critical need to understand the underlying mechanisms that determine tumor response to TMZ. We recently reported that chemoresistance to TMZ is related to a remodeling of the entire electron transport chain, with significant increases in the activity of complexes II/III and cytochrome c oxidase (CcO). Moreover, pharmacologic and genetic manipulation of CcO reverses chemoresistance. Therefore, to test the hypothesis that TMZ-resistance arises from tighter mitochondrial coupling and decreased production of reactive oxygen species (ROS), we have assessed mitochondrial function in TMZ-sensitive and -resistant glioma cells, and in TMZ-resistant glioblastoma multiform (GBM) xenograft lines (xenolines). Maximum ADP-stimulated (state 3) rates of mitochondrial oxygen consumption were greater in TMZ-resistant cells and xenolines, and basal respiration (state 2), proton leak (state 4), and mitochondrial ROS production were significantly lower in TMZ-resistant cells. Furthermore, TMZ-resistant cells consumed less glucose and produced less lactic acid. Chemoresistant cells were insensitive to the oxidative stress induced by TMZ and hydrogen peroxide challenges, but treatment with the oxidant L-buthionine-S,R-sulfoximine increased TMZ-dependent ROS generation and reversed chemoresistance. Importantly, treatment with the antioxidant N-acetyl-cysteine inhibited TMZ-dependent ROS generation in chemosensitive cells, preventing TMZ toxicity. Finally, we found that mitochondrial DNA-depleted cells (ρ°) were resistant to TMZ and had lower intracellular ROS levels after TMZ exposure compared with parental cells. Repopulation of ρ° cells with mitochondria restored ROS production and sensitivity to TMZ. Taken together, our results indicate that chemoresistance to TMZ is linked to tighter mitochondrial coupling and low ROS production, and suggest a novel mitochondrial ROS-dependent mechanism underlying TMZ-chemoresistance in glioma. Thus, perturbation of mitochondrial functions and changes in redox status might constitute a novel strategy for sensitizing glioma cells to therapeutic approaches.

## Introduction

Mitochondria are cellular organelles that play central roles in energy metabolism, retrograde signaling, and apoptosis. Within the mitochondria, oxidative phosphorylation (OxPhos) couples ATP production to the flow of electrons through the electron transport chain (ETC). Electron flow through the ETC occurs by the transfer of electrons through a chain of protein substrates (complexes I-IV). Upon reduction of the substrates, protons are released and a proton gradient is generated across the inner mitochondrial membrane that serves as the electrochemical energy gradient necessary for ADP phosphorylation. Low electron flux through the ETC can promote increased generation of superoxide radicals (O_2_^−^), particularly at complexes I and III [1], [2]. It has been reported that O_2_^−^ is generated constantly during cellular metabolism [3] and can be converted to hydrogen peroxide (H_2_O_2_) and other reactive oxygen species (ROS). Mitochondria are considered the major source of cellular ROS, which are intimately associated with retrograde signaling and affect many cellular functions [4]–[6]. Most cancer cells are under oxidative stress associated with increased metabolic activity and production of high levels of ROS. ROS generation may enhance the neoplastic behavior of a tumor by increasing genetic instability and the capacity to invade host tissues [7]. Although increased ROS production plays an important role in maintaining a cancer phenotype due to stimulatory effects on cell growth and proliferation [8], high levels of ROS can also cause cellular damage including lipid peroxidation, DNA and protein oxidation, and enzyme inactivation [5]. In general, cancer cells are metabolically active, produce high levels of ROS, and are under intrinsic oxidative stress, so they are more vulnerable to further oxidative stress by exogenous ROS-generating agents [5], [7], [9]–[13]. In particular, and relevant to this study, it was recently demonstrated that TMZ induces ROS production in gliomas as a result of DNA-damage [14].

The importance of mitochondria in tumor biology was revealed in Warburg's original studies. He postulated that the origin of cancer was due to irreversible damage to mitochondrial function, leading to enhanced glycolysis [15]–[17]. It has been suggested that the mitochondrial injury in cancer cells leads to a lower coupling efficiency of mitochondrial electron transport and ATP production, along with increased electron leakage and ROS generation during respiration, converting the mitochondrial respiration chain in cancer cells to a potential target for cancer therapy [5].

We hypothesized that the TMZ-tolerance of chemoresistant cells derives from tighter mitochondrial coupling and reduced ROS production. To test this hypothesis, we investigated the mitochondrial oxidative capacity of glioma cells and the ability of TMZ to induce oxidative stress. For these studies, we used a TMZ-resistant strain of U251 glioma cells (UTMZ), derived by selection for resistance to stepwise increases of TMZ concentrations. The ETC is remodeled dramatically in these TMZ-resistant cells; in particular, the activity of complex I is reduced and the activities of complexes II-III and cytochrome c oxidase (CcO or complex IV) are increased [18]. We also used different cellular models of mitochondrial dysfunction (ρ° cells, transmitochondrial cybrids and CcO knockout cells) and pharmacological approaches to investigate the role of mitochondrial ROS (mtROS) on the modulation of TMZ sensitivity.

## Results

### TMZ-resistant glioma cells have higher respiratory rates and tighter mitochondrial coupling

**Figure 1:**
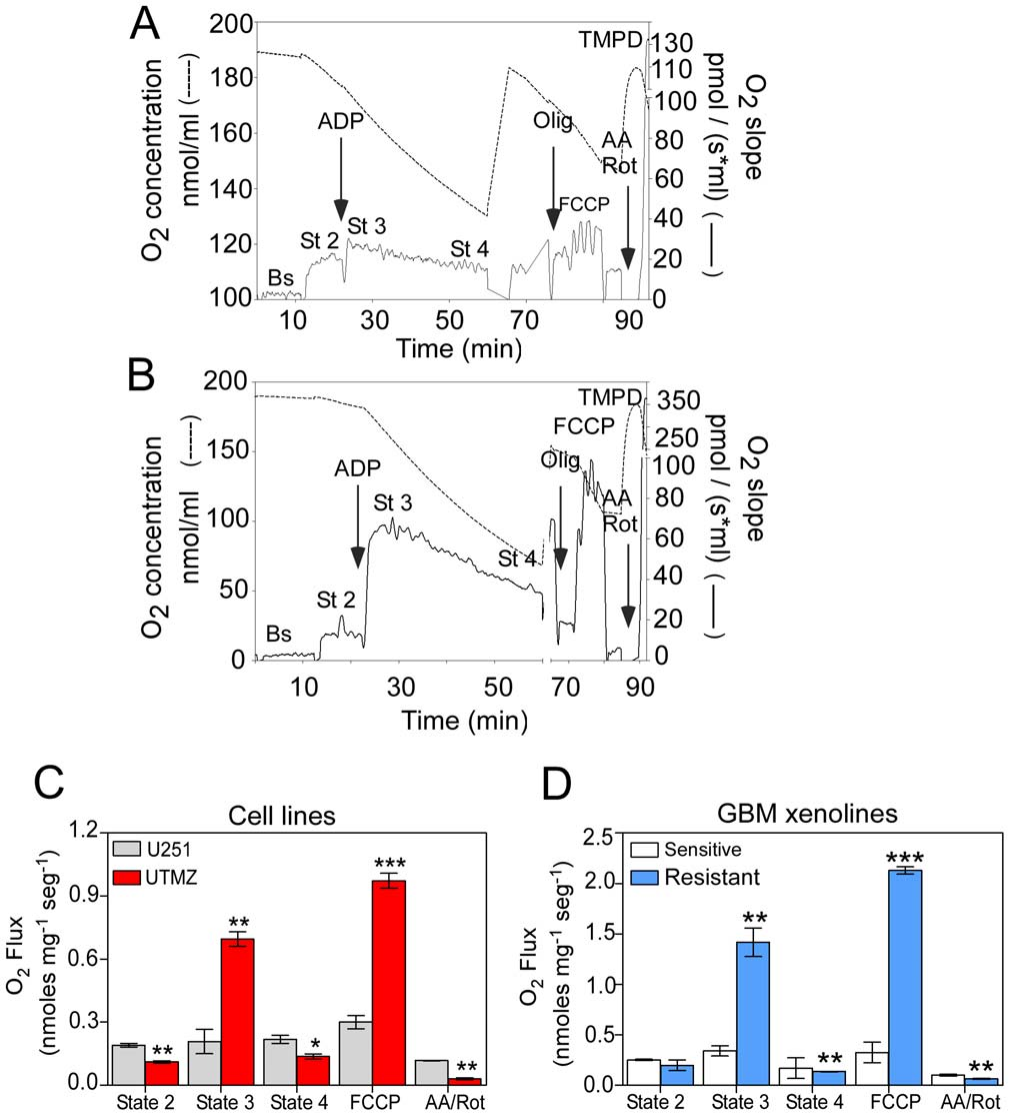
TMZ-resistant cells show a higher mitochondrial coupling. Oxygen consumption rates of isolated mitochondria (200 µg) were determined using a respirometer. Graphic illustrations of sample traces of mitochondrial respiration rates of (A) U251 and (B) UTMZ cells (dotted line, oxygen concentration; solid line, oxygen consumption). (C) Kinetic characterization of glutamate/malate-dependent respiration of isolated mitochondria from U251 and UTMZ cells. (D) Kinetic characterization of glutamate/malate-dependent respiration of isolated mitochondria from TMZ-sensitive and resistant GBM xenolines. Data are expressed as means ± S.E. of three independent experiments. *p*<0.05 (*), *p*<0.01 (**) and *p*<0.001 (***).

As an index of the mitochondrial functional capacity in TMZ-resistant and –sensitive cells, we measured the rate of oxygen (O_2_) consumption in intact mitochondria treated with 10 mM glutamate and 5 mM malate (complex I substrates) (Fig. 1A and B). The respiratory control ratio (RCR) (State 3/State 4) and acceptor control ratio ACR_olig_ (State 3/State 4i; the respiratory control ratio calculated after inhibiting ATP synthase using oligomycin [State 4i]) were measured as indicators of mitochondrial coupling. The RCR values were 4.59±0.96 and 1.29±0.12 for UTMZ and U251 cells, respectively. The ACR_olig_ value was 4.72±0.97 for UTMZ cells and 1.38±0.20 for U251 cells. Estimates were always greater for mitochondria from UTMZ cells, indicating that these mitochondria were highly coupled compared with mitochondria from the parental, TMZ-sensitive cells. The comparatively lower performance of mitochondria from U251 cells was due mainly to the decline of state 3 respiration, but the increase of states 2 and 4 respiration contributed, as well (Fig. 1A and C).

Maximal electron flux through the ETC was measured in the presence of the protonophore carboylcyanide-4-(trifluoromethoxy)-phenylhydrazone (FCCP), which causes dissipation of the proton gradient by carrying protons across the inner mitochondrial membrane. Dissipation of the proton gradient by FCCP leads to a rapid depolarization of mitochondria and acceleration of the flux of electrons through the transport chain. This resulted in significantly higher mitochondrial oxidative capacity in UTMZ cells. Inhibition of complexes I and III with rotenone and antimycin A, respectively, indicate a higher non-mitochondrial respiration in U251 compared to UTMZ cells (Fig. 1C).

Previously, we described a panel of serially transplantable GBM xenograft lines established by direct subcutaneous injection of patient tumor tissue in the flank of nude mice [18]. To distinguish these tumors from those induced in immunocompromised mice using established tumor cell lines maintained in culture, we have designated these as “xenolines.” To evaluate the clinical significance of our findings, we assessed the function and capacity of mitochondria from TMZ-sensitive and -resistant xenolines. The TMZ-resistant xenoline mitochondria have a higher maximum ADP-stimulated (State 3) O_2_ consumption rate and decreased respiration in states 2 and 4 (lower proton leak) when compared with the TMZ-sensitive line (Fig. 1D). RCR (5.74±0.56 vs. 1.35±0.15) and ACR_olig_ (10.46±0.69 vs. 2±0.45) indicated a tighter mitochondrial coupling in the TMZ-resistant xenograft line (Fig. 1D).

### TMZ-resistant cells show reduced glucose consumption and lactic acid production

**Figure 2:**
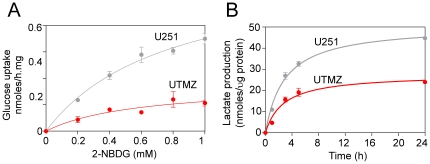
Glucose uptake and lactate production in U251-MG and UTMZ cells. (A) Dose-response analyses of glucose uptake. (B) Time course of lactate production. Data are shown as the means ± S.E. of two independent experiments performed in triplicate.

The high efficiency of mitochondrial metabolism (Fig. 1) in UTMZ cells might be reflected in a lower glycolytic rate. Figure 2A and B shows that both glucose consumption and lactate production are lower in UTMZ than in U251 glioma cells. Lineweaver-Burk and Eadie-Hofstee analysis yielded similar results. The Vmax for glucose consumption in UTMZ cells was significantly lower than the Vmax in U251 cells (Table 1). The Km for glucose in both cell lines was not significantly different. This Km is close to the expected Km for GLUT3 (Km<1 mM) [19], [20], and is consistent with our previous report indicating that U251 express GLUT3 on the cell surface [19], and might suggest that both cell lines express similar glucose transporters on their cell surface. Further, the rate of lactate formation was nearly 2-fold lower in the UTMZ cells than in the U251 cells (Fig. 2B). These results suggest that UTMZ are less glycolytic than the TMZ-sensitive U251 cells, indicating less of the Warburg effect.

**Table 1:** Kinetic analysis of glucose uptake in TMZ-sensitive and TMZ-resistant glioma cells.

	Vmax (nmoles/h/mg)		Km (mM)	
	U251	UTMZ	U251	UTMZ
Lineweaver-Burk	1.064±0.077	0.350±0.06^a^	1.05±0.075	1.11±0.038^b^
Eadie-Hofstee	1.176±0.12	0.376±0.01^a^	1.13±0.065	1.32±0.026^b^

Results are expressed as mean ± SE. (n = 3 separate determinations).

a p<0.01;

b p value no significative. Statistical analysis was performed using Student's two-tailed t test.

### TMZ-resistant cells exhibit decreased ROS production

**Figure 3:**
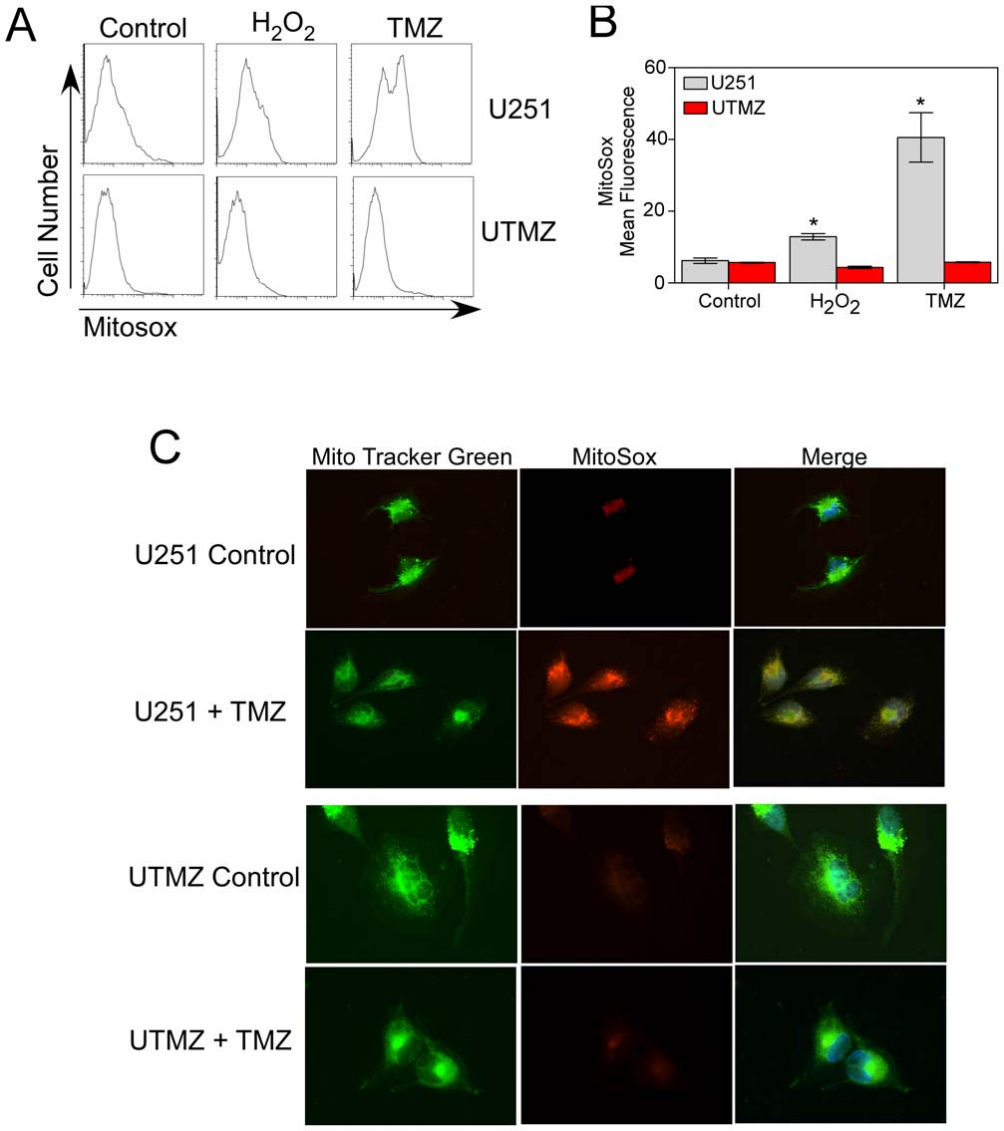
Determination of mitochondrial superoxide production. ROS production was estimated by oxidation of the fluorescent probe, MitoSox in TMZ-sensitive (U251) and TMZ-resistant (UTMZ) cells after 2 h of treatment with vehicle (control), H_2_O_2_ (100 µM) or TMZ (300 µM). (A) Representative histograms of flow cytometric analysis of MitoSox fluorescence. (B) Columns represent the MitoSox-mean fluorescence. Data are expressed as means ± S.E. of three independent experiments. *p*<0.05 (*). (C) Representative images acquired with epifluorescence microscopy showing co-localization of Mito Tracker Green and MitoSox fluorescence in U251 (Top panel) and UTMZ cells (Bottom panel).

The significant differences in state 2 and state 4 respiration in the mitochondria of these two cell models (Fig. 1) suggests possible changes in the production of ROS. To examine this, we measured acute mitochondrial O_2_^−^ formation by fluorescence microscopy and flow cytometry using MitoSOX Red, a fluoroprobe that selectively detects O_2_^−^ in mitochondria of live cells [21]. Both analyses demonstrated that treatment with TMZ caused increases in mitochondrial steady-state levels of O_2_^−^ in U251 cells (Fig. 3A, B and C). Since MitoSOX Red is relatively insensitive to oxidation by H_2_O_2,_ H_2_O_2_^−^ or vehicle-treated cells were used as negative controls (Fig. 3A). Quantitative analysis indicated that the steady-state production of mitochondrial O_2_^−^ by UTMZ cells is substantially lower than that of U251 cells (Fig. 3A, B and C).

**Figure 4:**
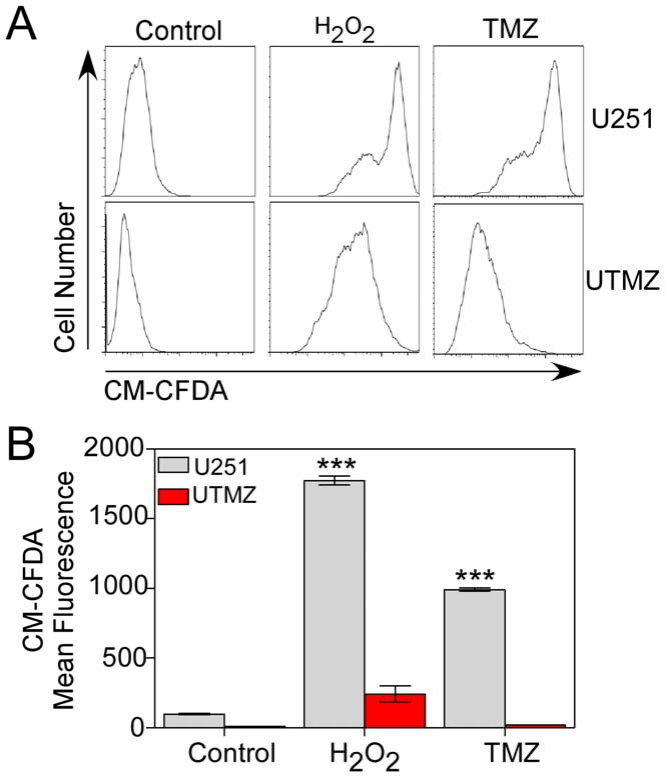
Determination of total ROS production. ROS production was estimated by oxidation of the fluorescent probe, CM-CFDA in TMZ-sensitive (U251) and TMZ-resistant (UTMZ) cells after 2 h of treatment with vehicle (control), H_2_O_2_ (100 µM) or TMZ (300 µM). (A) Representative histograms of flow cytometric analysis of MitoSox fluorescence. (B) Columns represent the CM-CFDA-mean fluorescence. Data are expressed as means ± S.E. of three independent experiments. *p*<0.001 (***).

In additional experiments, changes in total ROS production were estimated using CM-DCFDA, a cell-permeable probe that is non-fluorescent when chemically reduced but fluoresces after cellular oxidation and removal of acetate groups by cellular esterases. As shown in Figure 4A and B, H_2_O_2_ and TMZ challenges had no effect on the steady-state production of ROS by UTMZ cells, but increased total ROS production in U251 cells by 18-fold (H_2_O_2_) and 10-fold (TMZ). These results also indicate that the steady-state production of ROS by UTMZ cells is substantially lower than that of U251 cells.

Overall, the data from these studies (Fig. 3 and 4) indicate that treatment with pro-oxidants cause significant increases in intracellular steady-state levels of ROS (i.e., O_2_^−^ and H_2_O_2_) in U251 glioma cells. However, ROS generation by UTMZ cells was markedly lower. In addition, the studies using MitoSOX Red strongly support the hypothesis that mitochondria represent a major source for increased steady-state levels of O_2_^−^ in TMZ-sensitive glioma cells.

### TMZ-resistant cells exhibit decreased oxidative stress

**Figure 5:**
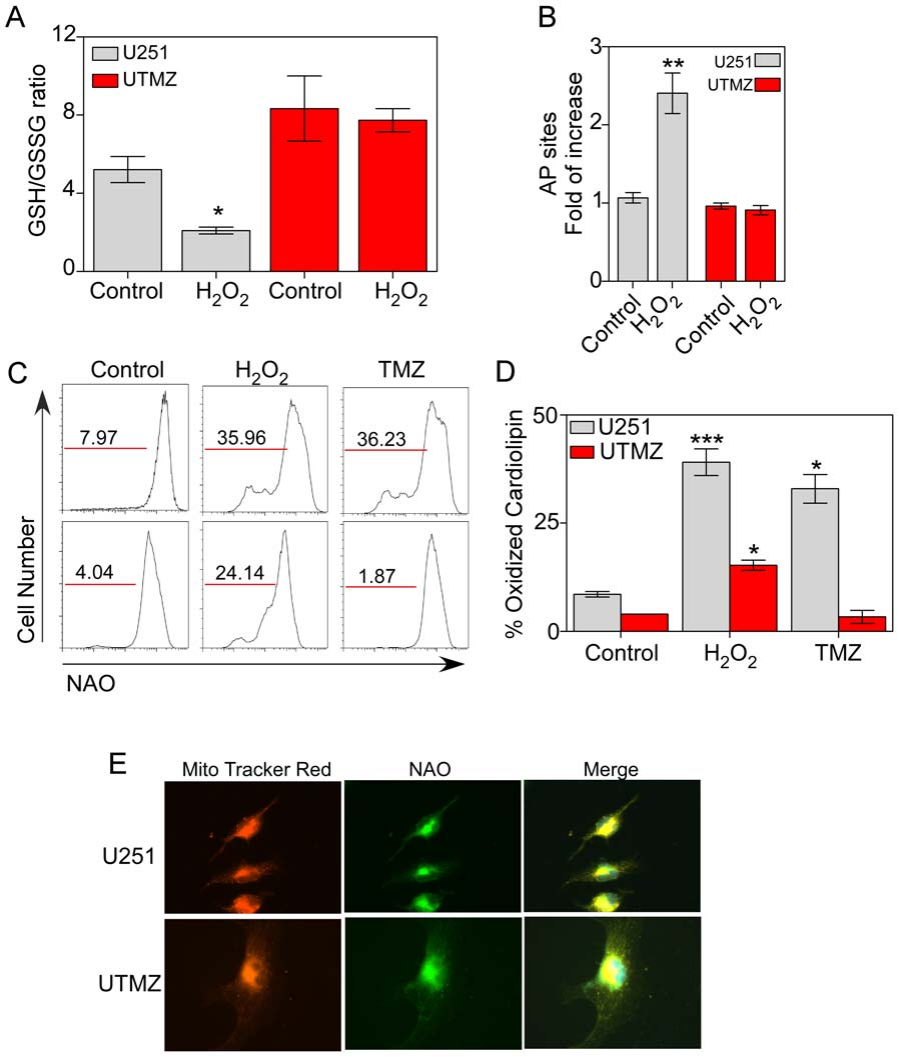
Determination of GSH/GSSG ratio, lipid peroxidation and AP sites generation. (A) GSH/GSSG ratio in U251 and UTMZ cells under control conditions or after treatment with 100 µM H_2_O_2_ for 1 h. (B) Generation of AP sites in mitochondrial DNA from U251 and UTMZ cells treated for 1 h with 100 µM H_2_O_2_. Columns represent the number of AP sites relative to the control. Data are expressed as means ± S.E. of three independent experiments. (C,D) Effect of H_2_O_2_ and TMZ on cardiolipin oxidation. (C) Representative histograms of flow cytometric analysis of NAO fluorescence, (D) Columns represent the NAO-mean fluorescence. Data are expressed as means ± S.E. of three independent experiments. *p*<0.05 (*), *p*<0.01 (**) and *p*<0.001 (***). (E) Representative images acquired with epifluorescence microscopy showing co-localization of Mito Tracker Red and NAO fluorescence in U251 (Top panel) and UTMZ cells (Bottom panel).

Reduced glutathione (GSH) is an intracellular anti-oxidant known to maintain cellular redox balance. We therefore measured intracellular GSH levels and determined the levels of oxidized glutathione (GSSG). H_2_O_2_ treatment did not produce a significant change in the GSH/GSSG ratio in TMZ-resistant UTMZ cells (Fig. 5A). However, there was a significant decrease in the GSH/GSSG ratio (2-fold) in U251 cells after exposure to 100 µM H_2_O_2_ for 1 h. Additionally, UTMZ cells had a significantly elevated (1.6-fold) GSH/GSSG ratio under basal conditions, suggesting an enhanced anti-oxidant capacity.

Mitochondrial lipid peroxidation, a downstream marker of oxidative damage, was examined using nonyl acridine orange (NAO) staining to detect oxidation of cardiolipin using flow cytometry. Cardiolipin is a membrane lipid component exclusively present in mitochondria that fluoresces yellow when labeled with NAO, [5], [22]. UTMZ cells were completely resistant to cardiolipin oxidation by TMZ, and H_2_O_2_-induced cardiolipin oxidation in UTMZ cells was significantly lower than in U251 cells treated either with TMZ or H_2_O_2_, as shown by a shift of NAO fluorescence towards the left (Fig. 5C, D and E).

To examine the differences in intracellular ROS production by U251 and UTMZ cells further, we measured the generation of apurinic/apyrimidinic (AP) sites in mitochondrial DNA (mtDNA) following treatment with 100 µM H_2_O_2_ for 1 h. AP sites are one of the major types of DNA lesions formed during the course of base excision and repair of oxidized, deaminated or alkylated bases [23], so the number of AP sites in cells is a good indicator of DNA lesion and repair against oxidative damage [23]. As shown in Figure 5B, UTMZ cells showed no significant change in generation of mitochondrial AP sites. In contrast, the number of mitochondrial AP sites increased significantly in U251 cells after H_2_O_2_ challenge.

### Mitochondrial ROS regulate TMZ-resistance

**Figure 6:**
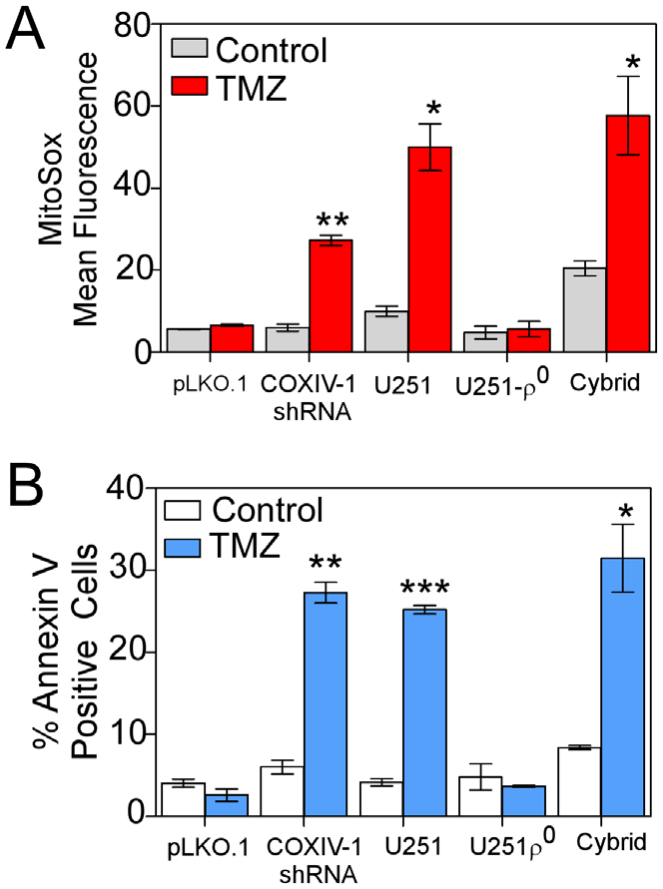
Determination of ROS production and apoptosis in cellular models of mitochondrial dysfunction. ROS production was estimated by oxidation of the fluorescent probes MitoSox in U251, U251-ρ° and U251-ρ° cybrid cells, and in COX-IV-1-shRNA transfected *versus* control, empty vector-transfected UTMZ cells (pLKO.1), in control vehicle-treated (control) cells or after 2 h of treatment with TMZ (300 µM). (A) Columns represent the MitoSox-mean fluorescence. (B) Quantification of apoptosis activation in different cellular models of mitochondrial dysfunction 72 h after treatment with TMZ (300 µM). Columns represent the average from triplicate determinations of the percentage of apoptotic cells. Data are expressed as means ± S.E. for three independent experiments. *p*<0.05 (*), *p*<0.01 (**) and *p*<0.001 (***).

Because UTMZ cells have a higher threshold for induction of O_2_^−^, we examined whether increased mitochondrial ROS would reverse TMZ resistance in chemoresistant glioma cells. Figure 6A shows mitochondrial ROS production in different cellular models. TMZ-resistant UTMZ cells were depleted of CcO (COXIV-1-shRNA) [18] and used as a model of decreased oxidative capacity. COXIV-1 shRNA cells exposed to 300 µM TMZ showed a significant increase in mitochondrial O_2_^−^ levels compared with empty vector-transfected UTMZ cells (pLKO.1) (Fig. 6A). Moreover, treatment with TMZ increased the percentage of annexin V-positive cells (apoptotic cells) in the COXIV-1 shRNA-transfected UTMZ cells, but did not affect the percentage of annexin V-positive cells in UTMZ cells transfected with the empty vector (pLKO.1) (Fig. 6B).

We also compared the effect of TMZ treatment on mtROS production using U251 cells, mtDNA-depleted U251 cells (U251 ρ° cells) and transmitochondrial cybrids [24] (U251-ρ° cells repopulated with parental (U251) mtDNA) (Fig. 6A). U251-ρ° cells were used as a genetic model of non-functional mitochondria. In the absence of TMZ, all isogenic cell lines generated small amounts of mtROS. TMZ-treatment did not increase ROS production in mtDNA-depleted U251-ρ° cells, but enhanced production of mtROS in the parental U251 cells and the trans-mitochondrial cybrids (Fig. 6A). After incubation with TMZ, the percentage of annexin V-positive cells increased in U251 and cybrid cell lines but not in U251-ρ° cells (Fig. 6B).

**Figure 7:**
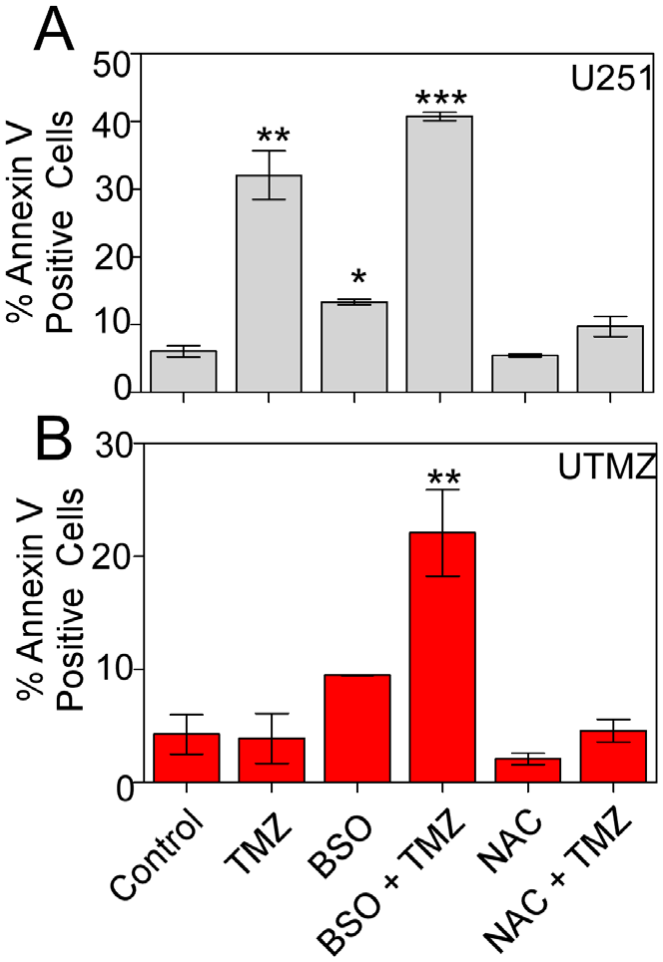
Effect of NAC and BSO on ROS production and TMZ-dependent apoptosis. (A) U251 and (B) UTMZ cells were treated with NAC (5 mM) or BSO (5 mM) for 24 h. Apoptotic cells were examined by Annexin V/Pi staining. Columns represent the average from triplicate determinations of the percentage of apoptotic cells. Data are presented as mean ± S.E. (n = 3). *p*<0.05 (*), *p*<0.01 (**) and *p*<0.001 (***)).

To test further whether ROS are involved in TMZ-dependent induction of apoptosis, U251 and UTMZ glioma cells were incubated with N-acetylcysteine (NAC), a thiol anti-oxidant, or L-buthionine-*S,R*-sulfoximine (BSO), an inhibitor of glutathione synthesis, and subsequently analyzed for TMZ-dependent apoptosis. As shown in Figure 7A, co-treatment with TMZ (300 µM) and BSO (5 mM) increased the percentage of apoptotic U251 cells compared with untreated or BSO-treated cells. Exposure of UTMZ cells to BSO did not induce a significant change in the percentage of apoptotic cells. However, co-treatment with TMZ and BSO caused a significant increase in the percentage of apoptotic cells. Next, we examined the effects of NAC (5 mM) on TMZ-dependent apoptosis. As shown in Figure 7A, NAC effectively prevented TMZ-induced apoptosis in U251 cells and had no additional effect in UTMZ cells. These findings demonstrate that perturbation of mitochondrial functions and changes in redox status are important components in the mechanism of TMZ-induced cell death.

## Discussion

GBM is the most frequent form of high grade malignant brain tumor. Since the study by Stupp et al. in 2005, the standard treatment for patients with primary GBM has included a regimen of concomitant and adjuvant chemotherapy with temozolomide (TMZ) [25], [26]. TMZ is an orally bioavailable alkylating agent that is known to target nuclear DNA and generate nuclear DNA adducts that block the cell cycle and lead to cell death through apoptosis. While the chemotherapeutic regimen is commonly used as part of the treatment against GBM and can be advantageous for short periods of time, chemotherapy is eventually impaired by the development of chemoresistance. This phenomenon represents the most challenging problem in the successful treatment of cancer and is the main reason for chemotherapy failure.

Our previous data showed a dramatic remodeling of the ETC in TMZ-resistant glioma cells. Of particular interest is the significant increase in CcO activity in the TMZ-resistant cell models and GBM patient biopsies taken after treatment and at the time of recurrence. We tentatively concluded that TMZ-resistant glioma cells, through an adaptive remodeling of the ETC activities, may have developed more efficient coupling between electron transport and ATP production, which enhanced the ability to increase mitochondrial electron transport during conditions of bioenergetic demand, conferring a selective advantage during the progression of the tumor. We proposed that modulation of the regulation of bioenergetics in the cancer cell plays an essential role in tumor progression after failure of chemotherapy [18].

Our present results indicate that chemoresistance to TMZ is due to more efficient mitochondrial coupling and reduced ROS production. Thus, we find that TMZ-resistant tumor cells under conditions of oxidative stress generate substantially less ROS, reduce states 2 and 4 of mitochondrial respiration, and overall, reduce cardiolipin oxidation and DNA oxidation as measured by the formation of AP sites in mtDNA. One possible explanation is decreased complex I activity and/or increased CcO activity [18]. Indeed, it is predicted that decreased activity of complex I combined with increased activity of complexes II-III and CcO may increase ETC efficiency, decrease mitochondrial proton and electron leak, and decrease the generation of mtROS. This may be particularly relevant as ROS are primarily generated at complex I of the ETC, with CcO not normally directly involved in ROS generation. Increased CcO activity would, however, confer an increased capacity for electron flux through the ETC which, in turn, would lead to an decreased ROS formation. This mechanism could be a part of a previously unrecognized adaptive chemoresistance mechanism that links both oxidative stress and drug resistance in cancer cells, leading to suppressed apoptotic signaling [27].

Our results point to tighter mitochondrial coupling and higher efficiency of mitochondrial metabolism in the UTMZ cells, as shown by an increase in state 3 respiration and an increase in the RCR and ACR ratios. Indeed, in these cells, both glucose consumption and lactate production are lower than in TMZ-sensitive U251 cells. To our knowledge, this is the first report of a bioenergetics profile of isolated chemoresistant mitochondria cancer cells. Together, our data pointed toward a better mitochondrial efficiency and a reversed “Warburg effect.” The coupling (or uncoupling) state of mitochondria is a key component of mitochondrial respiratory control. While resting (state 2) respiration was decreased in UTMZ cells, ADP-stimulated (state 3) coupled respiration was significantly increased to levels similar to those described in normal muscle and brain tissues [28], [29]. Importantly, TMZ-resistant GBM xenolines established directly from human GBM tumor tissues exhibited a similar respiration profile including tight mitochondrial coupling and high efficiency of mitochondrial metabolism.

Otto Warburg observed that cancer cells are often characterized by intense glycolysis in the presence of O_2_ and a concomitant decrease in mitochondrial [15]–[17]. While mitochondrial dysfunction has long been reported to contribute to cancer development, one possible explanation for our findings may be that cancer cells that survive a genotoxic stress can engage their “remodeled” ETC to survive. Due to the profound changes identified in the ETC of TMZ-resistant cells, we speculate that chemoresistant cells might result primarily from the selection of TMZ-resistant cells, in which ROS production was suppressed before initiation of the TMZ challenge, already existing in the original polyclonal population. However, the importance of clonal selection in the development of TMZ resistance of gliomas remains to be elucidated.

These results have led us to develop a central hypothesis that decreased glucose entry and lactate production can control the amount of reducing equivalents to the ETC, resulting in lower ROS production, particularly O_2_^−^, through the ETC. This regulatory mechanism at the level of the mitochondria may be extremely efficient and relevant in chemoresistant glioma cells, since genotoxic stressors such as DNA alkylating agents rely on ROS production to work [14], [30]. Additionally, the observation that FCCP greatly decreases ROS production suggests that anything that accelerates the movement of electrons down the ETC would decrease mtROS generation by depleting the electron-rich intermediates. In support of this, we previously showed that the activities of complexes II–III and CcO are substantially higher in UTMZ cells than in U251 cells [18]. Enhanced activities of these complexes might well cause depletion of electron-rich intermediates in the ETC in UTMZ cells.

Our data indicate that interference in the cellular redox balance can be exploited to overcome chemoresistance in glioma. We used ROS scavengers to eliminate the contribution of ROS to TMZ-induced apoptosis. Treatment with NAC greatly suppressed TMZ-induced apoptosis in U251 cells. On the other hand, in UTMZ cells that had an enhanced anti-oxidant capacity (GSH/GSSG ratio) after treatment with BSO, as compared to U251 cells, showed improvement in TMZ-sensitivity and apoptosis. Both of these data support the involvement of a redox-sensitive mechanism in chemoresistance.

Mitochondrial DNA-depleted (ρ°) mammalian cells and their cybrids have provided valuable cell models for studying important functions of mitochondria such as OxPhos, ATP production, electron transport, and ROS generation [24], [31]–[33]. ρ° cells lack a functional ETC and are incapable of generating ATP and ROS at the mitochondrial level [24], [34]. We reasoned that the non-functional mitochondria of U251-ρ° cells would affect the outcome of the cells after treatment with TMZ. Consistent with our hypothesis, U251-ρ° cells showed resistance to TMZ, and lower mitochondrial O_2_^−^ levels were also observed after TMZ treatment. When U251-ρ° cells were repopulated with mitochondria from U251 cells, these trans-mitochondrial cybrids recovered both their sensitivity to TMZ and ROS production. Overall, our results support the general idea that TMZ-sensitivity arises from enhanced production of ROS from electron-rich intermediates of the mitochondrial ETC. The TMZ-resistant UTMZ cells were found to have significantly less ROS production, and this was associated with enhanced chemoresistance. Accordingly, in our CcO knockout cellular model, besides the decrease in CcO activity and a parallel increase in the sensitivity to TMZ [18], we found an enhanced mtROS production under TMZ challenge.

**Figure 8:**
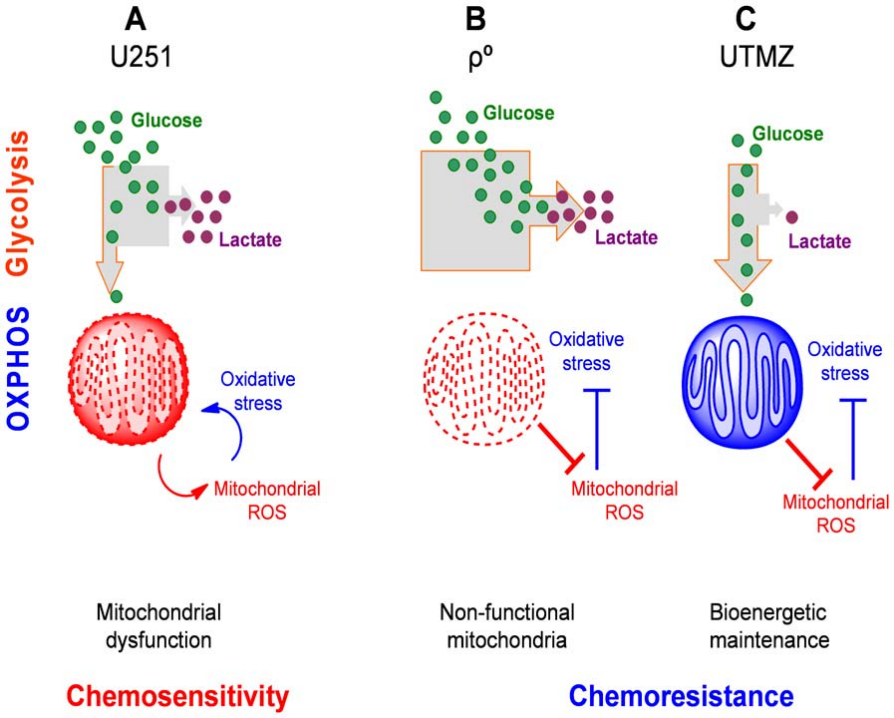
Mitochondrial regulation of chemosensitive and chemoresistance in glioma cells. (A) Glycolytic cells with functional OxPhos (Warburg effect) and high mtROS. (B) Exclusively glycolytic cells (Pasteur effect) and no mtROS production. (C) OxPhos cells with tight coupling and low mtROS.

Our data support an integrated model of chemoresistant and chemosensitive glioma where mitochondrial metabolism plays a central role (Fig. 8). In this model, glioma cells with a glycolytic phenotype but the ability to switch to OxPhos metabolism [19], [35]–[38] are sensitive to chemotherapy, and because they have dysfunctional mitochondria, mtROS is produced in excess. This endpoint is essential for compounds such as TMZ to be active (Fig. 8, Model A). Many anticancer drugs induce oxidative stress either as a direct mechanism of cell death or as an indirect effect of exposure, as observed with several chemotherapeutic agents [39]. Model B and C (Fig. 8) represent chemoresistant cells, with opposite bioenergetic phenotypes. Model B depicts cells that are exclusively glycolytic and unable to switch to OxPhos. Thus, these cells have low mtROS levels and illustrate more of the Pasteur Effect than the Warburg Effect demonstrated in Model A. An experimental model of these cells is represented by the mtDNA-depleted cancer cells used by us and other groups and described to be chemoresistant (Fig. 6) [40]–[42]. The opposite bioenergetic profile, illustrated in Model C, relies on a tight and efficient mitochondrial OxPhos pathway, which results from remodeling of the ETC, including decreased activity of Complex I and increased activities of Complexes II–III and CcO [18]. These cells, as demonstrated in this study, have low mtROS production and are resistant to oxidative stress. A similar mechanism has been previously reported for O_2_-tolerance in HeLa cells. Resistance to hyperoxia was found to be associated with increased mitochondrial efficiency and low mtROS production [43].

While Models B and C represent cells with opposite bioenergetic phenotypes, both have low mtROS levels, and consequently, both are chemoresistant. Indeed, pathways involved in the ROS-adaptive response could play a critical role in protecting cells against cytotoxic effects of anticancer agents, thus supporting the hypothesis of a correlation between adaptation to oxidative stress, low mtROS production and resistance to anticancer drugs [44]. Whether Models B and C describe pre-existing cells present within the bulk of the tumor (Model A) or cells that are transformed through chemotherapeutic treatment, the data presented here strongly indicate that chemoresistance in glioma is linked to low mtROS production and improved mitochondrial function. Moreover, combining pro-oxidant therapy with standard of care chemotherapy could be an effective strategy to overcome chemoresistance.

## Materials and Methods

### Cell culture

The human glioma cell line, U251, was kindly provided by Dr. Darell D. Bigner, Duke University, Durham, NC. TMZ-sensitive U251 cells, their TMZ-resistant counterparts (UTMZ) [18] and transmitochondrial cybrids [24] were grown in DMEM/F-12 medium plus L-glutamine supplemented with 7% heat-inactivated FBS, penicillin, and streptomycin. U251-ρ° cells [24] were cultured in DMEM/F12 supplemented with 7% FBS, 1 mM pyruvate and 50 µg/ml uridine. Cells were incubated at 37°C in a humidified atmosphere containing 5% CO_2_.

### Primary and TMZ-resistant GBM Xenograft Lines

The establishment and maintenance of the Mayo GBM xenograft panel has been described [45]. Briefly, TMZ resistance models were developed by subjecting mice with established flank tumors to successively higher doses of TMZ until tumor growth was no longer inhibited by 120 mg/kg/day TMZ for 5 days [44]. The resulting TMZ-resistant lines were maintained by serial passage. The efficacy of TMZ (66 mg/kg/day ×5 days) in orthotopic xenografts was evaluated as described [46].

### High resolution respirometry

Preparation of mitochondria was performed as previously described [18]. The integrity of the outer mitochondrial membrane was tested by addition of exogenous cytochrome c (cyt c) [47], [48]. The stimulatory effect of cyt c on O_2_ flux rates was lower than 5%, indicative of high quality mitochondrial preparations. Mitochondrial respiration assays using freshly isolated mitochondria were performed by measuring O_2_ consumption in a two-channel respirometer (Oxygraph-2k with DatLab software; Oroboros Instruments, Innsbruck, Austria) as previously described [47], [49]. Respiration rates were measured using a multisubstrate/inhibition protocol with saturating concentrations of substrates and inhibitors added consecutively. State 2 respiration was measured after the addition of 10 mM glutamate and 5 mM malate. Coupled respiration (state 3) was determined after the addition of 1 mM ADP, and state 4 (respiration not coupled to ATP synthesis) respiration was measured after the consumption of ADP. An induced state 4 (state 4i) respiration rate was established after the addition of 2 µg/ml oligomycin, followed by evaluation of maximal ETC capacity with a titration of the chemical uncoupler carbonylcyanide-4-(trifluoromethoxy)-phenylhydrazone (FCCP). Next, the following additions were made: 1 µM rotenone to inhibit the electron flow from complex I, 10 µM antimycin A to inhibit electron flow from complex II to cyt c, and 0.5 mM tetramethylphenylene diamine (TMPD) with 2 mM ascorbate to activate CcO.

### Glucose uptake

Uptake experiments were carried out as previously described [19] using 2-(*N*-(7-nitrobenz-2-oxa-1,3-diazol-4-yl)amino)-2-deoxyglucose (2-NBDG) (Invitrogen, Carlsbad, CA).

### Lactate production

Lactate accumulation in the culture medium was determined using a fluorescence-lactate assay kit from Cayman (Ann Arbor, MI), according to the manufacturer's instructions.

### Estimation of ROS production

Intracellular ROS production was determined by measuring the levels of O_2_^−^ and H_2_O_2_ produced in the cells by flow cytometry after staining the cells with CM-CFDA or MitoSOX™ Red (Invitrogen, Carlsbad, CA) as previously described [50]. Briefly, 0.3×10^6^ cells were plated in each well of six well plates and allowed to attach overnight, then exposed to either H_2_O_2_ or TMZ for varying time periods. Cells were further incubated with MitoSOX™ Red (2 µM) or CM-CFDA (5 µM) at 37°C for 25 min. Subsequently, cells were removed, washed and resuspended in PBS and analyzed for ROS production using a flow cytometer. Approximately 20,000 cells were evaluated for each sample. In all determinations, cell debris and clumps were excluded from the analysis.

### Imaging of NAO and MitoSox fluorescence

Cells were cultured on 12 mm glass cover slides. After treatment with vehicle or TMZ (300 µM, 2 h), cells were incubated with MitoSOX Red (2 µM) or NAO (0.2 µM) for 15 min and subsequently incubated with MitoTracker (0.2 µM) for 15 min before being imaged. Cells were washed and mounted in ProLong® Gold antifade reagent with DAPI (Invitrogen) and viewed with a Leica/Leitz DMRB fluorescence microscope equipped with appropriate excitation and emission filter sets (Chromatechnology). Images were acquired with a C5810 series digital color camera (Hamamatsu Photonic System). Images were processed with Corel® PaintShop Photo® Pro X3 Ultimate.

### Determination of apoptosis

The apoptosis-inducing effects of TMZ in U251, UTMZ, U251-ρ^0^, transmitochondrial cybrids, and COXIV-1-shRNA cells were determined by flow cytometery using annexin-V/7AAD as described previously [18]. About 0.1×10^6^ cells were plated in each well of 12-well plate and treated with 300 µM TMZ for 72 h. Apoptosis was determined using the PE Annexin V Apoptosis Detection Kit (BD Pharmigen), according to manufacturer's instructions, and analyzed by flow cytometry using a FACSCalibur (Becton Dickinson Biosciences) and FlowJo software from Tree Star.

### Estimation of AP sites in mtDNA

AP sites of DNA lesions were analyzed as we described previously [51]. Mitochondrial and genomic DNA were purified as we previously described [18], [24]. The number of AP sites was assessed using a DNA damage quantification kit (Abcam, Cambridge, MA) according to the manufacturer's instructions.

### Determination of oxidative damage to mitochondrial membrane

Mitochondrial membrane lipid peroxidation was detected by measuring the oxidation of intracellular cardiolipin, using 10-*N*-nonyl Acridine Orange (NAO) (Molecular Probes), a probe specific for mitochondrial membrane cardiolipin [5]. Briefly, cells were incubated for 1–3 h with 100 µM H_2_O_2_ or 300 µM TMZ, washed and then incubated with 2 µM NAO for 25 min at room temperature. After being washed with PBS, the cells were analyzed by flow cytometry. Approximately 20,000 cells were evaluated for each sample, and forward scatter versus side scatter was used to gate the viable population of cells. In all determinations, cell debris and clumps were excluded from the analysis.

### Determination of GSH and GSSG levels

GSH and GSSG levels were determined in glioma cells using a Bioxytech GSH/GSSG-412 kit according to the instructions of the manufacturer (OxisResearch, Portland, OR) as we described previously [50].

### Statistics

Data were evaluated using GraphPad. All reported *p* values are two-sided *t-test*, and *p* values of less than 0.01 were considered to indicate statistical significance. Experiments were performed in triplicate and were performed twice or more to verify the results. Data are shown as the means ± S.E. *p*<0.05 (*), *p*<0.01 (**) and *p*<0.001 (***).

## References

[1] Saybasili H, Yuksel M, Haklar G, Yalcin AS (2001). Effect of mitochondrial electron transport chain inhibitors on superoxide radical generation in rat hippocampal and striatal slices.. Antioxid Redox Signal.

[2] Staniek K, Gille L, Kozlov AV, Nohl H (2002). Mitochondrial superoxide radical formation is controlled by electron bifurcation to the high and low potential pathways.. Free Radic Res.

[3] Boveris A, Chance B (1973). The mitochondrial generation of hydrogen peroxide. General properties and effect of hyperbaric oxygen.. Biochem J.

[4] Richter C, Gogvadze V, Laffranchi R, Schlapbach R, Schweizer M (1995). Oxidants in mitochondria: from physiology to diseases.. Biochim Biophys Acta.

[5] Trachootham D, Zhou Y, Zhang H, Demizu Y, Chen Z (2006). Selective killing of oncogenically transformed cells through a ROS-mediated mechanism by beta-phenylethyl isothiocyanate.. Cancer Cell.

[6] Krzywanski DM, Moellering DR, Fetterman JL, Dunham-Snary KJ, Sammy MJ (2011). The mitochondrial paradigm for cardiovascular disease susceptibility and cellular function: a complementary concept to Mendelian genetics.. Lab Invest.

[7] Szatrowski TP, Nathan CF (1991). Production of large amounts of hydrogen peroxide by human tumor cells.. Cancer Res.

[8] Hu Y, Rosen DG, Zhou Y, Feng L, Yang G (2005). Mitochondrial manganese-superoxide dismutase expression in ovarian cancer: role in cell proliferation and response to oxidative stress.. J Biol Chem.

[9] Toyokuni S, Okamoto K, Yodoi J, Hiai H (1995). Persistent oxidative stress in cancer.. FEBS Lett.

[10] Toyokuni S (1998). Oxidative stress and cancer: the role of redox regulation.. Biotherapy.

[11] Kondo S, Toyokuni S, Iwasa Y, Tanaka T, Onodera H (1999). Persistent oxidative stress in human colorectal carcinoma, but not in adenoma.. Free Radic Biol Med.

[12] Devi GS, Prasad MH, Saraswathi I, Raghu D, Rao DN (2000). Free radicals antioxidant enzymes and lipid peroxidation in different types of leukemias.. Clin Chim Acta.

[13] Hileman EA, Achanta G, Huang P (2001). Superoxide dismutase: an emerging target for cancer therapeutics.. Expert Opin Ther Targets.

[14] Zhang WB, Wang Z, Shu F, Jin YH, Liu HY Activation of AMP-activated protein kinase by temozolomide contributes to apoptosis in glioblastoma cells via p53 activation and mTORC1 inhibition.. J Biol Chem.

[15] Warburg O (1956). On respiratory impairment in cancer cells.. Science.

[16] Warburg O (1956). [Origin of cancer cells].. Oncologia.

[17] Warburg O, Wind F, Negelein E (1927). The Metabolism of Tumors in the Body.. J Gen Physiol.

[18] Oliva CR, Nozell SE, Diers A, McClugage SG, Sarkaria JN (2010). Acquisition of temozolomide chemoresistance in gliomas leads to remodeling of mitochondrial electron transport chain.. J Biol Chem.

[19] Griguer CE, Oliva CR, Gillespie GY (2005). Glucose metabolism heterogeneity in human and mouse malignant glioma cell lines.. J Neurooncol.

[20] Medina RA, Meneses AM, Vera JC, Guzman C, Nualart F (2004). Differential regulation of glucose transporter expression by estrogen and progesterone in Ishikawa endometrial cancer cells.. J Endocrinol.

[21] Mukhopadhyay P, Rajesh M, Hasko G, Hawkins BJ, Madesh M (2007). Simultaneous detection of apoptosis and mitochondrial superoxide production in live cells by flow cytometry and confocal microscopy.. Nat Protoc.

[22] Pramanik KC, Boreddy SR, Srivastava SK (2011). Role of mitochondrial electron transport chain complexes in capsaicin mediated oxidative stress leading to apoptosis in pancreatic cancer cells.. PLoS One.

[23] Atamna H, Cheung I, Ames BN (2000). A method for detecting abasic sites in living cells: age-dependent changes in base excision repair.. Proc Natl Acad Sci U S A.

[24] Griguer CE, Oliva CR, Gobin E, Marcorelles P, Benos DJ (2008). CD133 is a marker of bioenergetic stress in human glioma.. PLoS One.

[25] Hegi ME, Diserens AC, Gorlia T, Hamou MF, de Tribolet N (2005). MGMT gene silencing and benefit from temozolomide in glioblastoma.. N Engl J Med.

[26] Stupp R, Mason WP, van den Bent MJ, Weller M, Fisher B (2005). Radiotherapy plus concomitant and adjuvant temozolomide for glioblastoma.. N Engl J Med.

[27] Landriscina M, Maddalena F, Laudiero G, Esposito F (2009). Adaptation to oxidative stress, chemoresistance, and cell survival.. Antioxid Redox Signal.

[28] Boushel R, Gnaiger E, Schjerling P, Skovbro M, Kraunsoe R (2007). Patients with type 2 diabetes have normal mitochondrial function in skeletal muscle.. Diabetologia.

[29] Lai JC, Clark JB (1976). Preparation and properties of mitochondria derived from synaptosomes.. Biochem J.

[30] Chandra J, Samali A, Orrenius S (2000). Triggering and modulation of apoptosis by oxidative stress.. Free Radic Biol Med.

[31] King MP, Attardi G (1989). Human cells lacking mtDNA: repopulation with exogenous mitochondria by complementation.. Science.

[32] Liu X, Kim CN, Yang J, Jemmerson R, Wang X (1996). Induction of apoptotic program in cell-free extracts: requirement for dATP and cytochrome c.. Cell.

[33] Li P, Nijhawan D, Budihardjo I, Srinivasula SM, Ahmad M (1997). Cytochrome c and dATP-dependent formation of Apaf-1/caspase-9 complex initiates an apoptotic protease cascade.. Cell.

[34] Weinberg F, Hamanaka R, Wheaton WW, Weinberg S, Joseph J (2010). Mitochondrial metabolism and ROS generation are essential for Kras-mediated tumorigenicity.. Proc Natl Acad Sci U S A.

[35] Griguer CE, Oliva CR, Gillespie GY, Gobin E, Marcorelles P (2007). Pharmacologic manipulations of mitochondrial membrane potential (DeltaPsim) selectively in glioma cells.. J Neurooncol.

[36] Bouzier AK, Voisin P, Goodwin R, Canioni P, Merle M (1998). Glucose and lactate metabolism in C6 glioma cells: evidence for the preferential utilization of lactate for cell oxidative metabolism.. Dev Neurosci.

[37] Bouzier AK, Goodwin R, de Gannes FM, Valeins H, Voisin P (1998). Compartmentation of lactate and glucose metabolism in C6 glioma cells. A 13c and 1H NMR study.. J Biol Chem.

[38] Turcotte ML, Parliament M, Franko A, Allalunis-Turner J (2002). Variation in mitochondrial function in hypoxia-sensitive and hypoxia-tolerant human glioma cells.. Br J Cancer.

[39] Tiligada E (2006). Chemotherapy: induction of stress responses.. Endocr Relat Cancer.

[40] Ferraresi R, Troiano L, Pinti M, Roat E, Lugli E (2008). Resistance of mtDNA-depleted cells to apoptosis.. Cytometry A.

[41] Park SY, Chang I, Kim JY, Kang SW, Park SH (2004). Resistance of mitochondrial DNA-depleted cells against cell death: role of mitochondrial superoxide dismutase.. J Biol Chem.

[42] Yu T, Yang Y, Liu S, Yu H (2009). Ultrasound increases DNA damage attributable to cisplatin in cisplatin-resistant human ovarian cancer cells.. Ultrasound Obstet Gynecol.

[43] Campian JL, Gao X, Qian M, Eaton JW (2007). Cytochrome C oxidase activity and oxygen tolerance.. J Biol Chem.

[44] Pennington JD, Wang TJ, Nguyen P, Sun L, Bisht K (2005). Redox-sensitive signaling factors as a novel molecular targets for cancer therapy.. Drug Resist Updat.

[45] Giannini C, Sarkaria JN, Saito A, Uhm JH, Galanis E (2005). Patient tumor EGFR and PDGFRA gene amplifications retained in an invasive intracranial xenograft model of glioblastoma multiforme.. Neuro Oncol.

[46] Carlson BL, Grogan PT, Mladek AC, Schroeder MA, Kitange GJ (2009). Radiosensitizing effects of temozolomide observed in vivo only in a subset of O6-methylguanine-DNA methyltransferase methylated glioblastoma multiforme xenografts.. Int J Radiat Oncol Biol Phys.

[47] Gnaiger E, Kuznetsov AV (2002). Mitochondrial respiration at low levels of oxygen and cytochrome c.. Biochem Soc Trans.

[48] Puchowicz MA, Varnes ME, Cohen BH, Friedman NR, Kerr DS (2004). Oxidative phosphorylation analysis: assessing the integrated functional activity of human skeletal muscle mitochondria–case studies.. Mitochondrion.

[49] Lanza IR, Nair KS (2009). Functional assessment of isolated mitochondria in vitro.. Methods Enzymol.

[50] Griguer CE, Oliva CR, Kelley EE, Giles GI, Lancaster JR (2006). Xanthine oxidase-dependent regulation of hypoxia-inducible factor in cancer cells.. Cancer Res.

[51] Zhou M, Zhao Y, Ding Y, Liu H, Liu Z (2010). Warburg effect in chemosensitivity: targeting lactate dehydrogenase-A re-sensitizes taxol-resistant cancer cells to taxol.. Mol Cancer.

